# *Bifidobacterium longum* BL‐19 inhibits oxidative stress and inflammatory damage in the liver of mice with NAFLD by regulating the production of butyrate in the intestine

**DOI:** 10.1002/fsn3.4279

**Published:** 2024-06-18

**Authors:** Xiajun Zhang, Jingwen Xu, Xueyun Dong, Jiajun Tang, Yan Xie, Jie Yang, Limin Zou, Liang Wu, Jilong Fan

**Affiliations:** ^1^ Department of Laboratory Medicine The People's Hospital of Danyang Zhenjiang Jiangsu China; ^2^ Department of Cardiology, Jurong Hospital Affiliated to Jiangsu University Zhenjiang Jiangsu China; ^3^ Department of Laboratory Medicine, School of Medicine Jiangsu University Zhenjiang Jiangsu China; ^4^ Hepatobiliary Surgery Lianyungang Second People's Hospital Affiliated to Jiangsu University Lianyungang China

**Keywords:** *Bifidobacterium longum* BL‐19 (*B. longum* BL‐19), butyric acid, CYP7A1, inflammation, nonalcoholic fatty liver (NAFLD), oxidative stress

## Abstract

Nonalcoholic fatty liver disease (NAFLD) is a common chronic liver disease, but there is currently no effective treatment method. Probiotics have been used as an adjunct therapy for NAFLD, but the mechanism is not clear. This study used *Bifidobacterium longum* BL19 (BL‐19) to treat the NAFLD mice induced by a high‐fat diet, and explored the treatment mechanism through gut microbiota and serum metabolomics techniques. We found that BL‐19 effectively prevented rapid weight gain in NAFLD mice and reduced their overall food and energy intake, decreased liver inflammatory factors expressions, and increased the bile acid synthetase enzyme CYP7A1 and superoxide dismutase. After BL‐19 treatment, the abundances of butyric acid bacteria (*Oscillospira* and *Coprococcus*) in the feces of mice increased significantly, and the concentration of butyric acid also increased significantly. We believe that BL‐19 promotes the production of butyrate in the intestines, which in turn regulates the activity of CYP7A1 in the liver and bile acid synthesis, ultimately treating liver inflammation and lipid accumulation in NAFLD mice. Serum metabolomics results indicated that BL‐19 affected multiple pathways related to inflammation and lipid metabolism in NAFLD mice. These findings suggest that BL‐19 shows promise as an adjunct therapy for NAFLD, as it can significantly improve oxidative stress, reduce inflammation in the liver, and decrease lipid accumulation.

## Graphical abstract

*Bifidobacterium longum* BL‐19 shows promise as an adjunct therapy for NAFLD, as it can significantly improve oxidative stress, reduce inflammation in the liver, and decrease lipid accumulation. Furthermore, *B. longum* BL‐19 enhances the production of butyric acid in the mouse gut. This increase in butyric acid regulates liver CYP7A1 activity and bile acid production, effectively treating inflammation and lipid deposition in the liver of NAFLD mice.

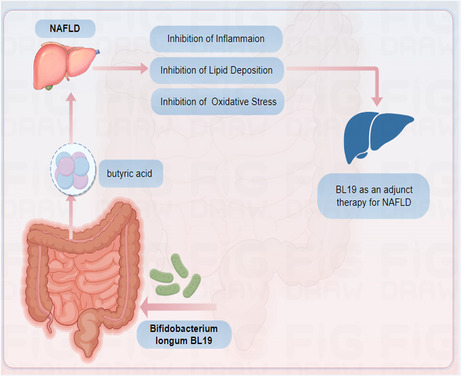

## 1 INTRODUCTION

Nonalcoholic fatty liver disease (NAFLD) is closely associated with insulin resistance, gut microbiota, and genetic susceptibility (Chiang, 2013; Loomba et al., 2021; Muzurović et al., 2021). If left untreated, simple fatty liver can progress to liver fibrosis, cirrhosis, and liver cancer (Tanwar et al., 2020). It is estimated that by 2030, the prevalence of NAFLD in the global population aged 15 and above could reach 33.5% (Teng et al., 2023). The pathogenesis of NAFLD is not fully understood, but the “multiple hits” hypothesis is widely accepted (Tilg et al., 2021). This hypothesis suggests that various factors, such as dietary patterns, insulin resistance, lipotoxicity, proinflammatory factors, and gut microbiota, contribute to the development of NAFLD (Zhang et al., 2024). Imbalanced diets can disrupt the gut microbiota, leading to chronic inflammation and increased intestinal permeability (Amabebe et al., 2020). Sustained low‐grade inflammation may play a crucial role in the progression from simple hepatic steatosis to fatty liver hepatitis and liver fibrosis (Salehi‐Sahlabadi et al., 2021).

Another factor that induces intestinal flora disorder should not be ignored. The widespread use of antibiotics not only in humans but also in animals has led to the presence of residues in derived foods, such as milk and dairy products. Recently, it has been emphasized that antibiotic‐induced changes in microbial composition reduce microbial diversity and alter the functional attributes of the microbiota. These antibiotic residues impact human gut flora, setting in motion a chain of events that leads straight to various metabolic alterations that can ultimately contribute to the onset and progression of NAFLD (Tarantino & Citro, 2024).

Clinical doctors have utilized probiotic‐based approaches as adjunctive treatments for NAFLD (Liang et al., 2023). Commonly used probiotics include *Lactobacillus*, *Streptococcus*, and *Bifidobacterium*. It is noted that several studies have demonstrated the cholesterol‐lowering and blood lipid‐regulating effects of certain probiotics, including *Lactobacillus plantarum*, *Lactobacillus acidophilus*, *Bifidobacterium*, and *Lactobacillus rhamnosus*, as well as fermented products containing probiotics (Huang et al., 2020; Olas, 2020). Consuming these probiotics in appropriate amounts can effectively regulate blood lipid levels and significantly contribute to the prevention of various metabolic diseases (Li et al., 2020). Sarcopenia, closely linked to NAFLD, could be usefully approached by probiotics (Tarantino et al., 2023). Probiotics offer advantages such as high safety and minimal side effects, making them highly marketable as an adjunctive treatment option (Bustamante et al., 2020).

*Bifidobacterium longum* is a strain of Bifidobacteria that is commonly used and has been proven to effectively colonize the human gut in various trials. Once it establishes itself, *B. longum* positively influences the existing microbial community by increasing levels of propionic acid, biotin, and butyric acid, thereby impacting gut metabolism (Marras et al., 2021; Pham et al., 2021). However, there is limited research on its therapeutic effects on NAFLD. This study aims to explore the potential therapeutic effects of *B. longum* to NAFLD mice, offering a novel approach to the clinical treatment of NAFLD.

## 2 MATERIALS AND METHODS

### 2.1 NAFLD mice model and grouping

Male ICR mice weighing 27 g ± 3 g were obtained from Wukong Biotechnology Company in Nanjing, China and were housed at the Experimental Animal Center of Jiangsu University. The study consisted of three experimental groups: a normal control group (NC group), a NAFLD group, and a BL‐19 strain intervention group (BL group), with 12 mice in each group. The NAFLD and BL groups were fed a high‐fat diet (60% fat energy) to induce NAFLD (product number: D12109, Future Biotech, Beijing, China), while the NC group was fed a regular diet. The regular diet had a caloric value of 3.84 kcal/g, while the high‐fat diet had a caloric value of 5.24 kcal/g.

During the experiment, the average body weight of each group of mice was monitored every 7 days, and the feed consumption of each mouse was calculated at the end of the experiment. The *B. longum* BL‐19 strain used in the study was obtained from Zhongke‐Jiayi Company in Qingzhou, Shandong, China. The mice in the BL group were orally administered the *B. longum* BL‐19 strain (1 × 10^7^ CFU) daily until the end of the 12th week of the experiment. All three groups of mice were provided with sterile purified water and had unrestricted access to drink. At the conclusion of the experiment, serum, liver, and colon contents were collected from the mice and stored at −80°C for future experiments. Additionally, mouse liver samples were fixed in 10% formalin for 48 h and stained with hematoxylin and eosin (HE).

### 2.2 Measurement of serum biochemical indicators and feces butyric acid

The measurement of alanine aminotransferase (ALT), aspartate aminotransferase (AST), total bile acids (TBA), triglycerides (TG), total cholesterol (TC), total bile acids (TBA), superoxide dismutase (SOD), and malondialdehyde (MDA) in mouse serum was conducted using kits from Nanjing Jiancheng Bioengineering Institute (Nanjing, China). These kits were carefully tested in accordance with the provided instructions to ensure accurate results.

The content of butyric acid in mouse feces was detected using the HPLC–MS/MS method by Shanghai Labway Medical Testing Laboratory (Shanghai, China).

### 2.3 The qPCR assay

The quantitative PCR was used to detect the expression levels of inflammation and bile acid metabolism‐related genes in mouse liver. The primers were synthesized by GENEWIZ company (Suzhou, China) (Table 1.). The total volume of qRT‐PCR was 20 μL, with 20 μL of SYBR Green Master Mix (Vazyme, Nanjing, China), 0.4 μL (10 μmol/L) of upstream and downstream primers, and 2 μL of cDNA template. The reaction program was as follows: predenaturation at 95°C for 5 min, denaturation at 95°C for 5 s, annealing at 58°C for 20 s, extension at 72°C for 30 s, and a total of 40 reaction cycles for qPCR.

**TABLE 1:** The primer sequence of qPCR assay.

Genes	Primer sequences (5′ → 3′)
*Mum_GAPDH*	F: AATGGATTTGGACGCATTGGT
	R: TTTGCACTGGTACGTGTTGAT
*Mum_TNF‐α*	F: GAACTGGCAAAAGGATGGTGA
	R: TGTGGGTTGTTGACCTCAAAC
*Mum_NLRP3*	F: ATTACCCGCCCGAGAAAGG
	R: CATGAGTGTGGCTAGATCCAAG
*Mum_IL‐10*	F: GAAGCTCCCTCAGCGAGGACA
	R: TTGGGCCAGTGAGTGAAAGGG
*Mum_FXR*	F: GTGAGGGCTGCAAAGGTTTC
	R: CAAACCTGTATACATACATTCAGCC
*Mum_FGF‐15*	F: ACTGCGAGGAGGACCAAAAC
	R: GAGTAGCGAATCAGCCCGTA
*Mum_CYP7A1*	F: CCGATGGATGGAAATACCAC
	R: GGCAGCGGTCTTTGAGTTAG

### 2.4 Mouse liver HE staining and oil red O staining

Shanghai Labway Medical Laboratory (Shanghai, China) conducted HE staining and oil red O staining on mouse liver tissue. The liver tissue was fixed in 10% formalin for 48 h before undergoing HE staining. After fixation, the liver tissue was dehydrated using graded alcohols and embedded in paraffin for sectioning, resulting in sections with a thickness of 4 μm. The liver tissue morphology was then observed under a microscope following the completion of HE staining. For frozen sections of mouse liver tissue, samples were fixed in 4% formaldehyde‐phosphate buffer (pH 7.4) for 10 min, followed by rinsing with distilled water and 60% isopropanol. Subsequently, the samples were stained with 4% oil red O dye for 10 min. After washing with water and 60% isopropanol, the mouse liver tissue sections were stained with hematoxylin. The distribution of fat within the liver cells was observed under a microscope after staining.

### 2.5 Analysis of 16S rRNA gene sequencing of mouse colon contents

We collected the contents of the mouse colon and extracted bacterial genomic DNA from mouse feces using a bacterial genomic DNA extraction kit (TIANGEN, Beijing, China). The bacterial 16S rRNA gene sequencing analysis was conducted by ekemo Tech Group Co., Ltd. (Shenzhen, China). The sequencing was performed on the Illumina HiSeq high‐throughput sequencing platform, and the sequence analysis and species annotation were carried out using bioinformatics analysis. We further conducted various analyses, including OTU clustering analysis, α‐diversity analysis, and β‐diversity analysis.

### 2.6 Nontargeted metabolomic analysis of mouse serum

The method used to collect mouse serum samples for nontargeted metabolomic analysis was as follows: mouse serum was collected in a blood collection tube without anticoagulant and left at room temperature for 30 min to allow for serum separation. The separated serum was then centrifuged at 6000× *g* for 10 min to obtain serum for nontargeted metabolomic analysis. To prepare the samples, 200 μL of mouse serum was mixed with 600 μL of methanol by vortexing for 15 s. After centrifugation at 15,000× *g* for 15 min, the supernatant was taken for nontargeted metabolomic analysis. The nontargeted metabolomic analysis was conducted by ekemo Tech Group Co., Ltd. (Shenzhen, China). The LC–MS/MS data collected were subjected to peak extraction and matching, and the data of total peak area were normalized. Principal component analysis (PCA) and orthogonal partial least square‐discriminate analysis (OPLS‐DA) were performed using SIMCA 14.1 software. Metabolite selection was based on variable importance in projection (VIP) and *t*‐test (*p* < .05) using the Metabo Analyst 4.0 platform. Further clustering analysis and pathway analysis of the differentially expressed metabolites in the serum were conducted.

### 2.7 Statistical analysis

The data are presented in the format of mean ± standard deviation (SD), and statistical analysis is conducted using SPSS 23.0 statistical software. To determine significant differences between multiple groups of samples, single‐factor analysis of variance (ANOVA) and the least squares degeneracy (LSD) method are employed, with a significance level of *p* < .05. GraphPad Prism 8.0 software (Prism, San Diego, CA, USA) is utilized for data visualization.

## 3 RESULTS

### 3.1 BL‐19 strain reduced body weight and energy intake in NAFLD mice

**FIGURE 1:**
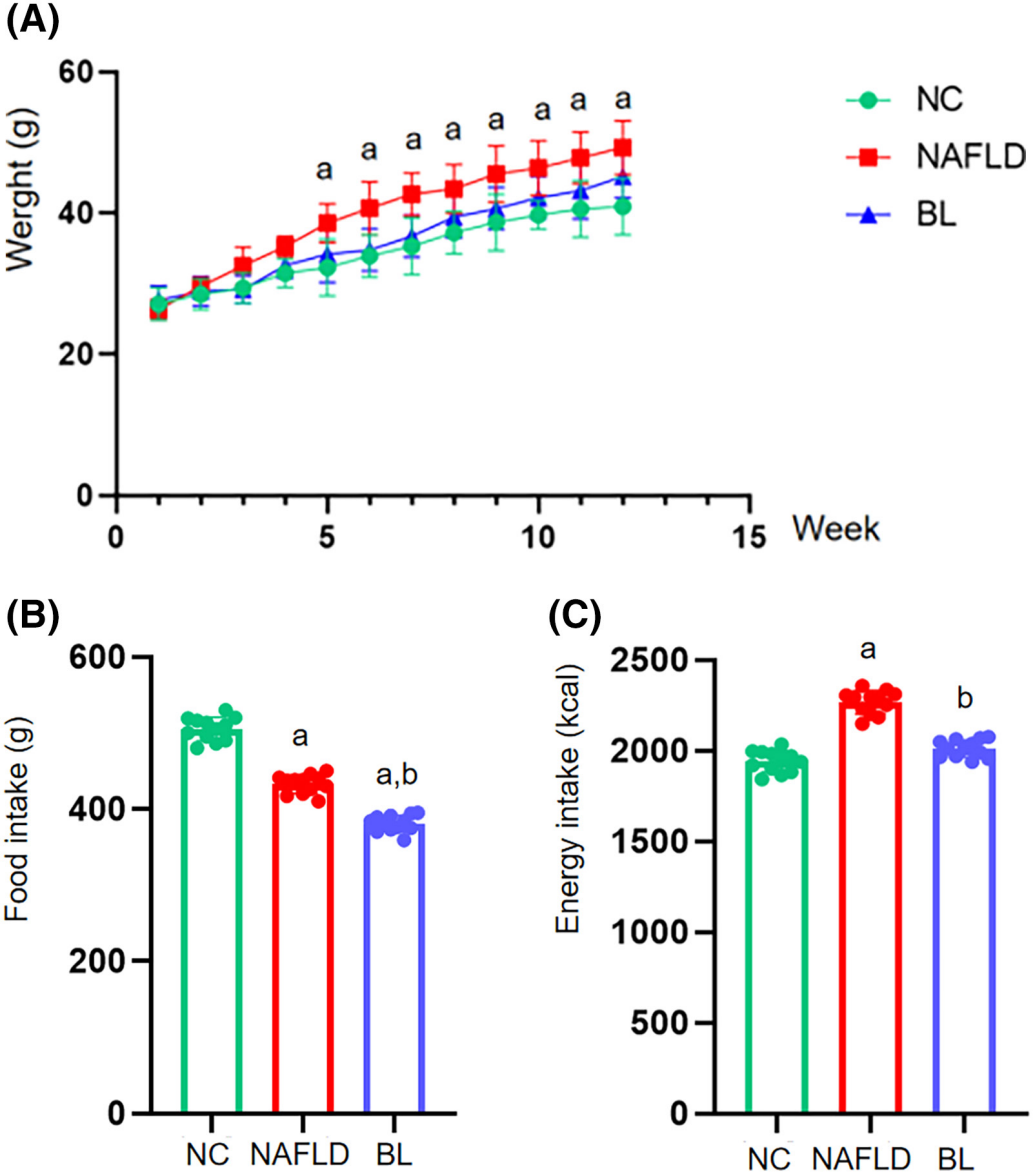
Body weight, feed intake, and total energy intake of mice. (A) body weight; (B) feed intake; (C) total energy intake. a: compared with NC group, *p* < .05; b: compared with NAFLD group, *p* < .05.

Starting from the 5th week, the mice in the NAFLD group had a significantly higher body weight compared to the mice in the NC group (*p* < .05). Throughout the entire experiment, there was no significant difference in body weight between the mice in the BL group and either the NAFLD group or the NC group (*p* > .05) (Figure 1A). Both the NAFLD group and the BL group mice had significantly decreased feed consumption compared to the NC group (*p* < .05). Additionally, the BL group mice had a significant reduction in feed consumption compared to the NAFLD group (*p* < .05) (Figure 1B). The energy intake of the mice in the NAFLD group was significantly higher than that of the mice in the NC and BL groups throughout the entire experiment (*p* < .05). However, there was no significant difference in energy intake between the NC and BL groups (*p* > .05) (Figure 1C).

### 3.2 BL‐19 regulates the inflammation and bile acid synthesis genes in liver

**FIGURE 2:**
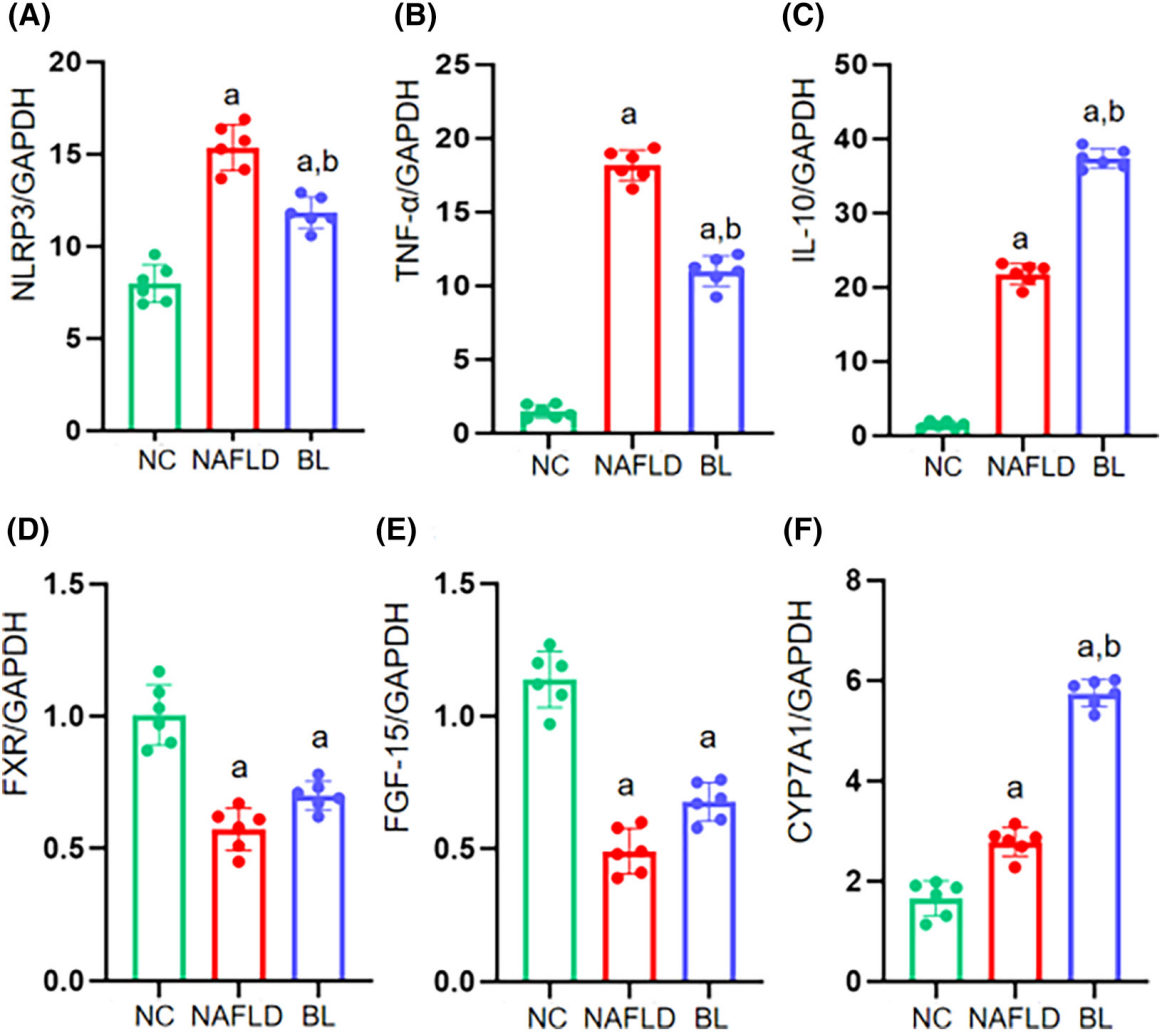
The expressions of inflammatory‐related factors including NLRP3, TNF‐α, IL‐10, and the expression of bile acid metabolism including FXR, FGF‐15, and CYP7A1 in mice. (A) NLRP3; (B) TNF‐α; (C) IL‐10; (D) FXR; (E) FGF‐15; (F) CYP7A1. a: compared with NC group, *p* < .05; b: compared with NAFLD group, *p* < .05.

Compared to the NC group, the levels of proinflammatory factors TNF‐α and NLRP3 in the liver tissues were significantly higher in both the NAFLD and BL groups (*p* < .05). However, the BL group showed a significant decrease in TNF‐α and NLRP3 expression levels compared to the NAFLD group (*p* < .05) (Figure 2A,B). Additionally, the levels of anti‐inflammatory cytokine IL‐10 in the liver tissues were significantly higher in both the NAFLD and BL groups compared to the NC group (*p* < .05). However, the BL group exhibited a significant decrease in IL‐10 expression levels compared to the NAFLD group (*p* < .05) (Figure 2C).

In comparison to the NC group, the liver tissues of the NAFLD and BL groups exhibited significant decreases in the expression levels of bile acid metabolism‐related factors FXR and FGF‐15, while the expression level of CYP7A1 increased (*p* < .05). When compared to the NAFLD group, only the expression level of CYP7A1 in the liver tissues of the BL group mice significantly increased (*p* < .05), with no significant changes observed in the expression levels of FXR and FGF‐15 (Figure 2D–F).

### 3.3 BL‐19 improved serum inflammatory and oxidative stress indexes and fecal butyric acid content in NAFLD mice

**FIGURE 3:**
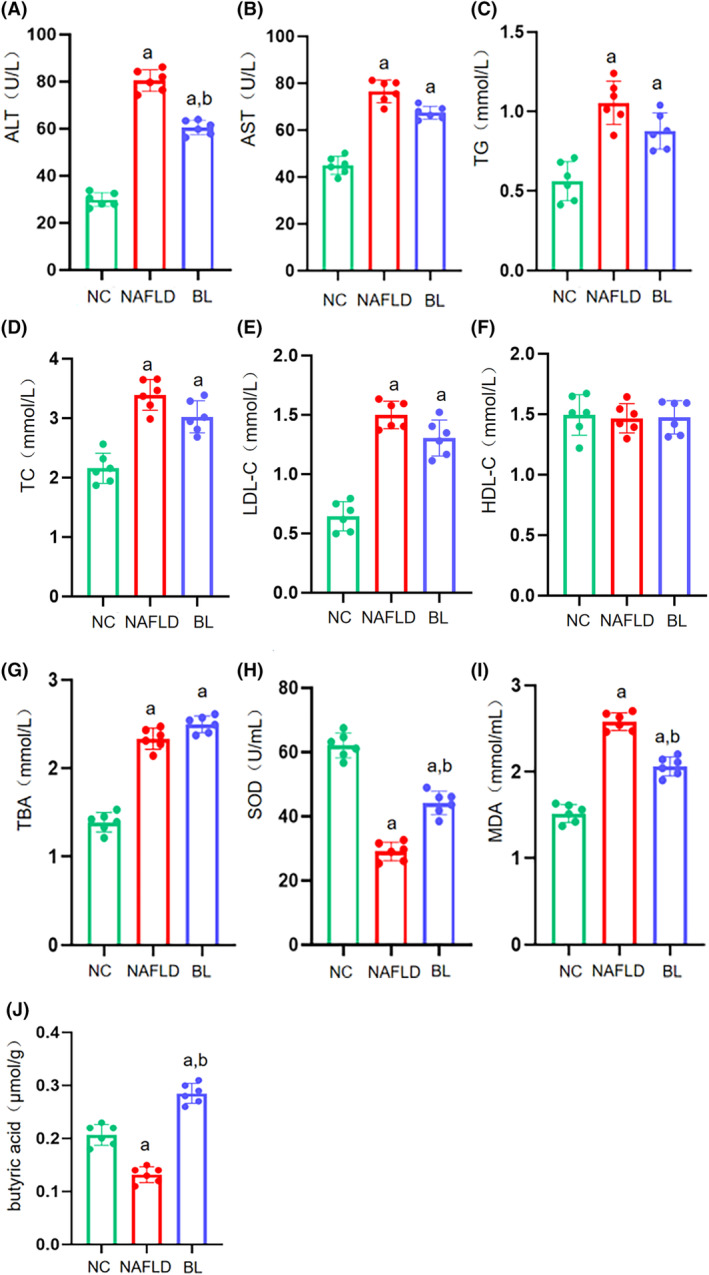
Serum biochemical indices and oxidative stress levels in mice. (A) ALT; (B) AST; (C) TG; (D) TC; (E) LDL; (F) HDL; (G) TBA; (H) SOD; (I) MDA; (J) butyric acid. a: compared with NC group, *p* < .05; b: compared with NAFLD group, *p* < .05.

Compared to the NC group, the NAFLD and BL groups showed significant increases in levels of ALT, AST, TC, TG, and LDL in the serum (*p* < .05). Only the ALT level in the BL group mice showed a significant decrease compared to the NAFLD group (*p* < .05), while other indicators showed no significant changes (*p* > .05). The serum HDL levels in all three groups of mice remained unchanged (*p* > .05) (Figure 3A–F).

Compared to the NC group, the serum levels of TBA were significantly higher in the NAFLD and BL groups (*p* < .05). Although the TBA level in the BL group mice seemed to be higher compared to the NAFLD group, the difference was not statistically significant (*p* > .05) (Figure 3G).

The serum levels of SOD were significantly lower in the NAFLD and BL groups compared to the NC group (*p* < .05). However, the BL group showed significantly higher SOD levels compared to the NAFLD group (*p* < .05) (Figure 3H). Conversely, the serum levels of MDA were significantly higher in the NAFLD and BL groups compared to the NC group (*p* < .05). However, the BL group showed significantly lower MDA levels compared to the NAFLD group (*p* < .05) (Figure 3I).

The butyric acid level in the feces of the NAFLD group mice was significantly lower compared to the NC group, but significantly higher in the BL group (*p* < .05). Additionally, the BL group mice had a significantly higher butyric acid level in their feces compared to the NAFLD group (*p* < .05) (Figure 3J).

### 3.4 BL‐19 alleviates liver damage and lipid deposition in NAFLD mice

**FIGURE 4:**
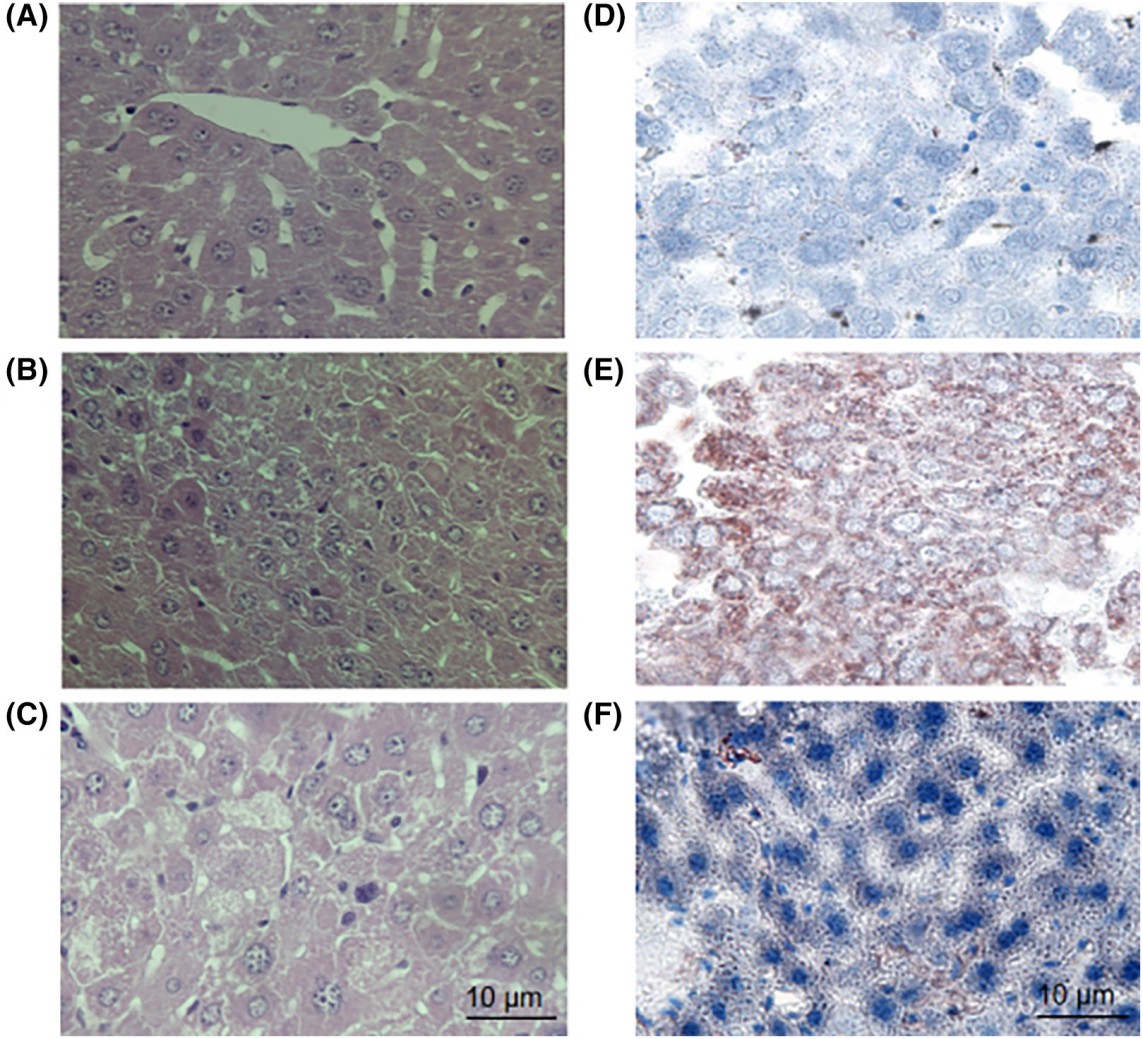
Mouse liver was stained with HE and oil red O. (A) HE staining of liver tissue in the NC group; (B) HE staining of liver tissues in the NAFLD group; (C) HE staining of liver tissue in the BL group; (D) Oil red O staining of liver tissue in the NC group; (E) Oil red O staining of liver tissue in the NAFLD group; (F) Liver tissue oil red O staining in the BL group.

According to the results of HE staining, the NC group exhibited a regular arrangement of hepatic lobules and cords, with no presence of inflammatory cells or visible fat vacuoles (Figure 4A). Conversely, the NAFLD group displayed a significantly irregular arrangement of hepatic lobules and cords, along with hepatocyte degeneration, swelling, and visible fat vacuoles (Figure 4B). In comparison to the NAFLD group, the BL group exhibited a decrease in inflammatory cells within liver cells, a reduction in fat infiltration, and a decrease in fat vacuoles (Figure 4C). Additionally, oil red O staining revealed no lipid accumulation in liver cells in the NC group (Figure 4D). In contrast, the NAFLD group displayed deeply stained and larger lipid droplets within liver cells (Figure 4E). In the BL group, the staining of liver cells was lighter, and the number and volume of lipid droplets were reduced (Figure 4F).

### 3.5 BL‐19 remodeled the intestinal flora of NAFLD mice

**FIGURE 5:**
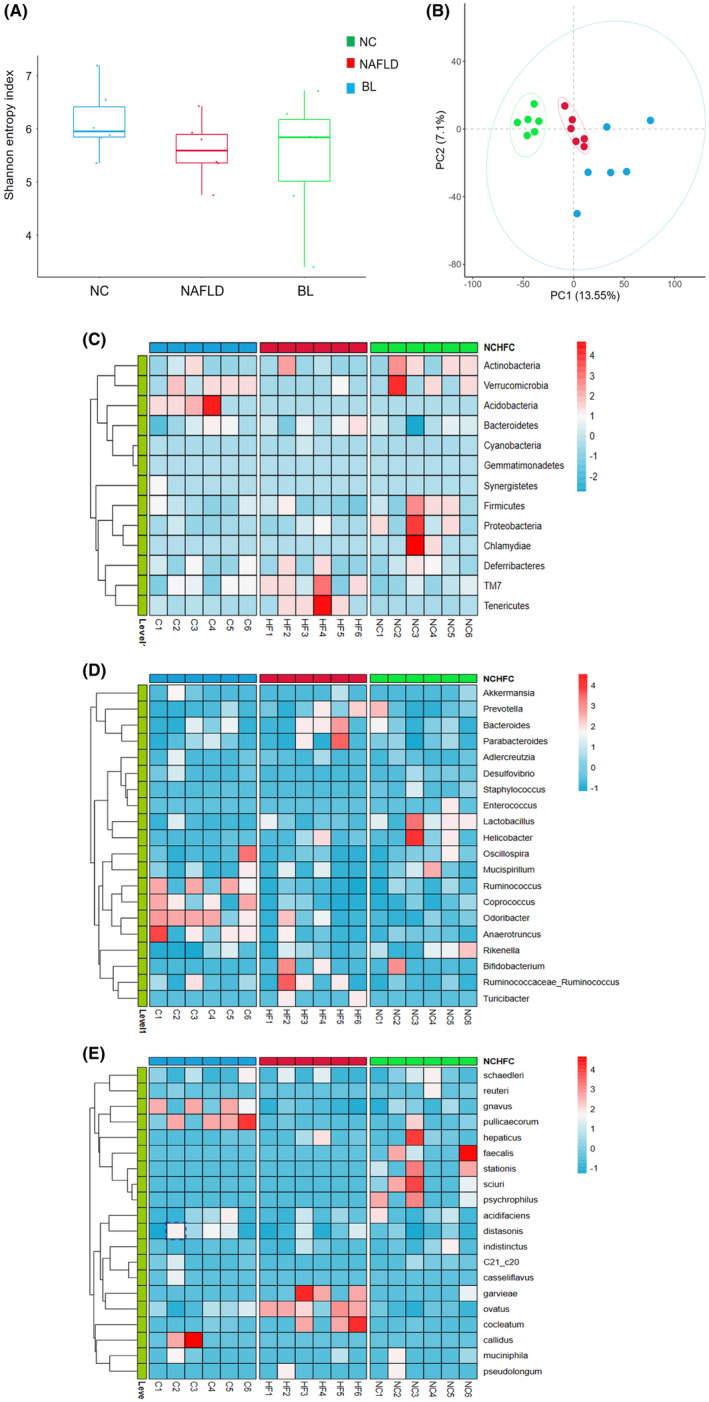
Results of intestinal flora analysis in mice. (A) α‐diversity (Shannon index); (B) β‐diversity (PCA scatter plot); (C) Clustering heat map in the phylum level; (D) Clustering heat map in the genus level; (E) Clustering heat map in the species level.

Using Illumina Hiseq high‐throughput sequencing technology, we obtained OTU sequences to examine the diversity and species composition of the intestinal microbiota in various groups of mice. Our goal was to assess the impact of *B. longum* BL‐19 on reshaping the intestinal microbiota in NAFLD mice. The α‐diversity analysis results indicated that there were no significant differences in the Shannon index among the three groups of mouse intestinal microbiota (*p* > .05) (Figure 5A).

The β‐diversity analysis results demonstrate a notable distinction and separate clustering between the samples from the NC and NAFLD groups, indicating a significant disparity in gut microbiota composition between NAFLD mice and normal mice. While the samples from the BL group also displayed significant separation from the NC and NAFLD groups, they may not necessarily cluster together. This finding suggests a substantial distinction in the gut microbiota composition of the BL group, but a significant difference remains in the gut microbiota composition among all three groups (Figure 5B).

We conducted a more detailed examination of the variations in the composition of gut microbiota among three different groups of mice, focusing on the phylum, genus, and species levels. In terms of phylum, we observed that the NAFLD group exhibited a noteworthy rise in the relative abundance of *TM7* and *Verrucomicrobia*, while the relative abundance of *Firmicutes* was significantly reduced compared to the NC group. However, following the administration of BL‐19, we observed a significant decrease in the relative abundance of *TM7* and *Verrucomicrobia*, accompanied by a substantial increase in the relative abundance of *Acidobacteria* and *Firmicutes* (Figure 5C).

Compared to the NC group, the NAFLD group exhibited a significant increase in the relative abundance of *Bacteroides*, *Parabacteroides*, and *Ruminococcus* at the genus level. Conversely, the relative abundance of *Lactobacillus* and *Spirochaetes* decreased. Following treatment with *B. longum* BL‐19, there was a decrease in the relative abundance of *Bacteroides*, *Parabacteroides*, and *Ruminococcus*, while *Anaerostipes*, *Fusobacterium*, and *Clostridium* showed a significant increase (Figure 5D).

At the level of species, the NAFLD group showed a significant increase in the relative abundance of *Geobacillus*, *Bacteroides*, and *Fusobacterium* compared to the NC group. Conversely, *Helicobacter*, *Enterococcus*, *Prevotella*, *Staphylococcus*, and *Pseudomonas* were significantly decreased in the NAFLD group. Following treatment with BL‐19, there was a significant decrease in the relative abundance of *Geobacillus*, *Bacteroides*, and *Fusobacterium*, while *Ruminococcus*, *Lactobacillus*, and *Ruminococcus* showed a significant increase (Figure 5E).

### 3.6 Results of nontargeted metabolomics analysis in mouse serum

**FIGURE 6:**
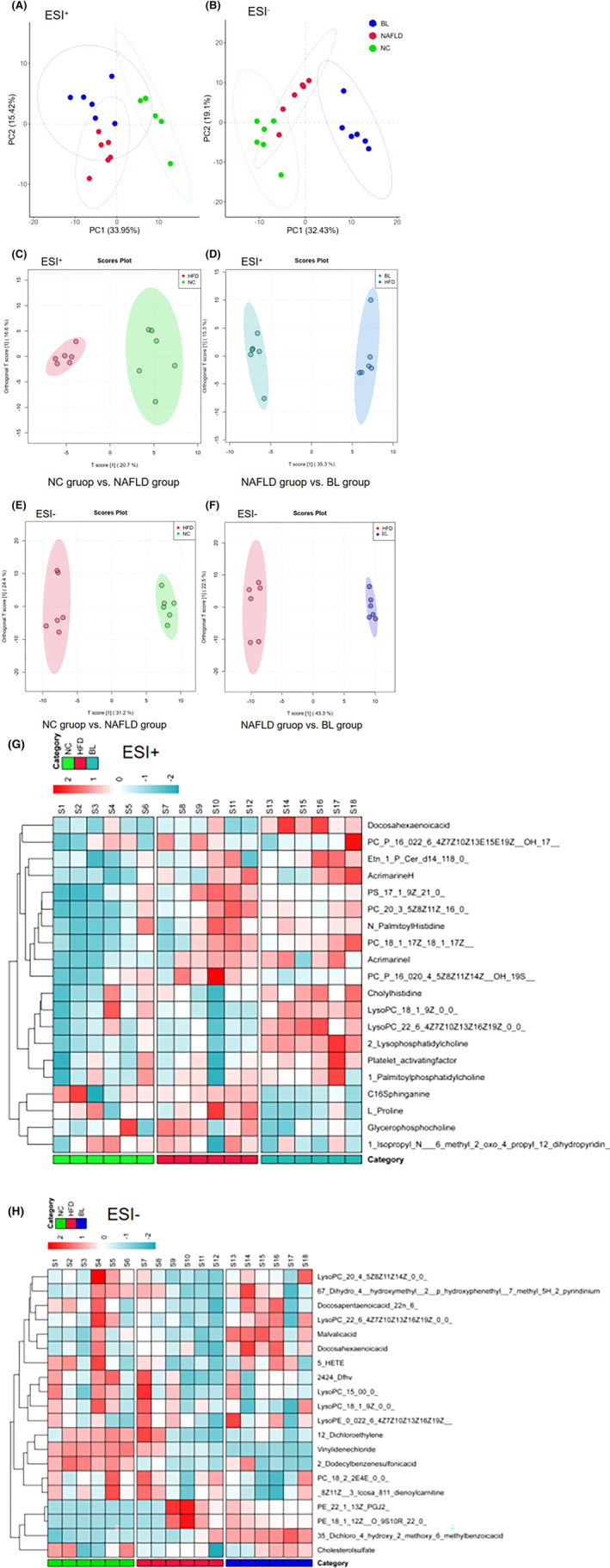
Results of serum metabolomics analysis in mice. (A) PCA scatter plot (ESI^+^); (B) PCA scatter plot (ESI^−^); (C) OPLS‐DA scatter plot (NC group vs. NAFLD group, ESI^+^); (D) OPLS‐DA scatter plot (NC group vs. NAFLD group, ESI^−^); (E) OPLS‐DA scatter plot (NAFLD group vs. BL group, ESI^+^); (F) OPLS‐DA scatter plot (NAFLD group vs. BL group, ESI^−^); (G) Clustering heat map of serum differential metabolites (ESI^+^); (H) Clustering heat map of serum differential metabolites (ESI^−^).

The PCA analysis of mouse serum metabolomics showed that the three groups of samples were significantly separated in both ESI^+^ and ESI^−^ modes, and the samples in each group clustered well in the ESI‐mode (Figure 6A,B). Further OPLS‐DA analysis showed that the three groups of serum samples clustered well and were significantly separated (Figure 6C–F). Based on the VIP > 1 and *p* < .05 criteria, differential metabolites were selected (Tables 2 and 3, Figure 6G,H). Compared with the NC group, the levels of inflammation and oxidative stress‐related factors Hypoxanthine, Galactinol, Adrenic acid, Heptadecanoic acid, and 2‐Dodecylbenzenesulfonic acid in mouse serum metabolites of the NAFLD group were significantly increased; the levels of proinflammatory lipid metabolites 1‐Palmitoyl phosphatidylcholine, LysoPC (20:4(8Z,11Z,14Z,17Z)/0:0), LysoPC (18:1(9Z)/0:0), and LysoPE (0:0/22:6(4Z,7Z,10Z,13Z,16Z,19Z)) were also increased. Compared with the NAFLD group, the levels of proinflammatory lipid metabolites 2‐Lysophosphatidylcholine, Palmitoylcarnitine, and Oleoylcarnitine in mouse serum metabolites of the BL group were significantly decreased, while the levels of anti‐inflammatory and antioxidative stress metabolites, indoleacrylic acid, glycerophosphocholine, uric acid, stearoylethanolamide, L‐carnitine, and 3‐O‐sulfogalactosylceramide (d18:1/20:0) were significantly increased.

**TABLE 2:** Serum differential metabolites of mice in the NC and NAFLD groups.

No.	Metabolite	Formula	HMDB	m/z	Retention time (min)	Which‐max
1	PC_P_16_018_1_12Z__2OH_910	C42H82NO9P	HMDB0289479	758.5688574	22.39506667	↓
2	Platelet‐activating factor	C26H54NO7P	HMDB0062195	546.353062	11.28525	↑
3	PE_P_18_0LTE4_	C46H83N2O10PS	HMDB0285308	909.5402457	22.40195	↓
4	PE_NMe2_15_022_5_7Z10Z13Z1	C44H78NO8P	HMDB0113936	780.551732	13.56821667	↓
5	Uric acid	C5H4N4O3	HMDB0000289	169.0356496	2.205783333	↑
6	PC_20_3_5Z8Z11Z_16_0_	C44H82NO8P	HMDB0008364	784.5825037	15.82271667	↓
7	DG_O_16_018_00_0_	C37H74O4	HMDB0011146	627.5319963	22.40195	↓
8	Cholesta_579_11__trien_3beta_ol	C27H42O	HMDB0250159	383.3291818	22.25763333	↑
9	Cholesterol‐5beta‐hydroperoxide	C27H46O3	HMDB0250177	383.3291867	21.42535	↑
10	Phenol glucuronide	C12H14O7	HMDB0060014	541.1553762	18.02981667	↓
11	PC_O_16_016_1_9Z__	C40H80NO7P	HMDB0013404	718.5711487	15.5546	↑
12	LysoPC_20_4_5Z8Z11Z14Z_0_0	C28H50NO7P	HMDB0010395	566.321933	8.6669	↑
13	1‐Palmitoyl phosphatidylcholine	C24H50NO7P	HMDB0256091	518.3219085	9.223533333	↑
14	Hypoxanthine	C5H4N4O	HMDB0000157	137.0457013	2.185183333	↑
15	Galactinol	C12H22O11	HMDB0005826	377.0852857	0.7602	↑
16	LysoPC(20:4(8Z,11Z,14Z,17Z)/0:0)	C28H50NO7P	HMDB0010396	588.3293491	10.39696667	↑
17	3‐O‐Sulfogalactosylceramide(d18:1/20:0)	C44H85NO11S	HMDB0012315	870.5483222	10.28015	↓
18	PE(20:0/18:3(9Z,12Z,15Z))	C43H80NO8P	HMDB0009227	790.5408607	10.87353333	↑
19	12_KETE	C20H30O3	HMDB0013633	317.211439	10.99723333	↓
20	16‐Hydroxyhexadecanoic acid	C16H32O3	HMDB0006294	317.2115701	11.61945	↓
21	(2′ E,4′Z,7′Z,8E)_Colnelenic acid	C18H28O3	HMDB0030996	629.4549201	12.93863333	↓
22	CPA(18:0/0:0)	C21H41O6P	HMDB0007004	457.2256215	14.33925	↓
23	DG(15:0/22:5(7Z,10Z,13Z,16Z,19Z)/0:0)	C40H68O5	HMDB0007091	627.4984601	13.79445	↓
24	LysoPC(17:0/0:0)	C25H52NO7P	HMDB0012108	554.3451641	11.52316667	↓
25	LysoPC(18:1(9Z)/0:0)	C26H52NO7P	HMDB0002815	566.3451239	11.05395	↑
26	LysoPC(20:5(5Z,8Z,11Z,14Z,17Z)/0:0)	C28H48NO7P	HMDB0010397	586.313423	9.762666667	↓
27	LysoPE(18:2(9Z,12Z)/0:0)	C23H44NO7P	HMDB0011507	476.277269	10.4382	↓
28	LysoPE(0:0/22:6(4Z,7Z,10Z,13Z,16Z,19Z))	C27H44NO7P	HMDB0011496	524.2771743	10.45195	↑
29	Kinetensin 4‐7	C26H37N9O6	HMDB0012986	592.2640484	10.45195	↑
30	LysoPC(20:4(5Z,8Z,11Z,14Z)/0:0)	C28H50NO7P	HMDB0010395	588.329452	10.55048333	↓
31	PE(22:5(7Z,10Z,13Z,16Z,19Z)/16:0)	C43H76NO8P	HMDB0009649	764.5268842	10.85291667	↑
32	Cholesterol sulfate	C27H46O4S	HMDB0000653	465.3038299	13.33911667	↑
33	Adrenic acid	C22H36O2	HMDB0002226	331.2633988	13.6364	↑
34	PC(18:0/P_16:0)	C42H84NO7P	HMDB0008060	780.5662762	15.48383333	↓
35	Heptadecanoic acid	C17H34O2	HMDB0002259	269.2474664	14.4011	↑
36	Allolithocholic acid	C24H40O3	HMDB0000381	357.27914	13.82195	↑
37	2‐Dodecylbenzenesulfonic acid	C18H30O3S	HMDB0031031	325.1835593	13.79445	↑
38	6,7‐Dihydro‐4‐(hydroxymethyl)‐2‐(p‐hydroxyphenethyl)‐7‐methyl‐5H‐2‐pyrindinium	C18H22NO2+	HMDB0033483	265.1468937	13.78758333	↑

**TABLE 3:** Serum differential metabolites of mice in the NAFLD and the BL groups.

No.	Metabolite	Formula	HMDB	m/z	Retention time (min)	Which‐Max
1	2‐Lysophosphatidylcholine	C26H54NO7P	HMDB0258493	524.3713425	10.8386	↓
2	1‐Stearoylglycerol	C21H42O4	HMDB0244009	341.3050936	16.64133333	↓
3	LysoPC_17_00_0	C25H52NO7P	HMDB0012108	510.3559962	10.15123333	↓
4	PC_P_16_020_4_5Z,8Z,11Z,14Z_OH_19S	C44H80NO8P	HMDB0289433	782.5674368	15.82958333	↑
5	PC_P_18_1_9ZPGJ2	C46H80NO9P	HMDB0289769	804.5498197	15.47225	↑
6	LysoPC_22_6_4Z,7Z,10Z,13Z,16Z,19Z0_0	C30H50NO7P	HMDB0010404	568.3399228	8.58445	↓
7	Indoleacrylic acid	C11H9NO2	HMDB0000734	188.0706996	4.4529	↑
8	Glycerophosphocholine	C8H20NO6P	HMDB0000086	258.1102437	0.900233333	↑
9	Uric acid	C5H4N4O3	HMDB0000289	169.0356496	2.205783333	↑
10	PC_22_6_4Z,7Z,10Z,13Z,16Z,19Z20_	C50H82NO8P	HMDB0008738	838.5774169	18.55208333	↑
11	L‐Proline	C5H9NO2	HMDB0000162	80.04910278	21.01291667	↑
12	DG_22_6_4Z,7Z,10Z,13Z,16Z,19Z20_2_	C45H72O5	HMDB0007776	675.5379387	17.31501667	↓
13	Stearoylethanolamide	C20H41NO2	HMDB0013078	310.3105902	16.49685	↑
14	PC_O_16_016_1_9Z	C40H80NO7P	HMDB0013404	718.5711487	15.5546	↑
15	LysoPC_18_2_9Z,12Z0_0	C26H50NO7P	HMDB0010386	542.3219489	8.625666667	↑
16	Palmitoylcarnitine	C23H45NO4	HMDB0000222	400.34191	9.02425	↓
17	1‐Palmitoylphosphatidylcholine	C24H50NO7P	HMDB0256091	518.3219085	9.223533333	↑
18	Oleoylcarnitine	C25H47NO4	HMDB0005065	426.3571322	9.333583333	↓
19	L‐Carnitine	C7H15NO3	HMDB0000062	162.1125045	0.96895	↑
20	L‐Tyrosine	C9H11NO3	HMDB0000158	146.0598752	4.4529	↑
21	Sphinganine	C18H39NO2	HMDB0000269	302.305486	7.388183333	↑
22	Dodecenoylcarnitine	C19H35NO4	HMDB0251567	342.2643001	5.800466667	↑
23	2‐Acetyloxy‐3‐octadecoxypropyl2‐trimethylazaniumylethylphosphate	C28H58NO7P	HMDB0242460	574.3835978	14.21456667	↓
24	4_[4_[_2R,5R_5_[2__Dibenzylamino_2_oxoethyl]_2_heptyl_4_oxo_1,3_thiazolidin_3_yl]butyl]benzoicacid	C37H46N2O4S	HMDB0252985	659.286707	14.18706667	↑
25	TG_15_014_1_9ZO_18_0	C50H96O5	HMDB0043191	815.6899579	14.86061667	↓
26	Mdo_npa	C20H21NO2	HMDB0254383	637.3052136	14.17333333	↑
27	3‐Palmitoyl‐sn‐glycerol	C19H38O4	HMDB0245964	313.2736158	14.1321	↓
28	Cer_d17_116_0	C33H65NO3	HMDB0240685	524.5014874	11.23715	↓
29	2_Oxa_4,7,12_triazatridecan_13_oicacid,9_hydroxy_5__1_methylethyl_3,6_dioxo_8,11_bis_phenylmethyl_1__2_pyridinyl_,3_pyridinylmethylester,_5S,8S,9S,11S_	C37H43N5O6	HMDB0244627	654.3303822	14.16645	↑
30	2‐Hydroxydocosanoylcarnitine	C29H57NO5	HMDB0241600	538.3854429	12.05543333	↓
31	Galactinol	C12H22O11	HMDB0005826	377.0852857	0.7602	↓
32	LysoPC(20:4(8Z,11Z,14Z,17Z)/0:0)	C28H50NO7P	HMDB0010396	588.3293491	10.39696667	↑
33	3‐O‐Sulfogalactosylceramide(d18:1/20:0)	C44H85NO11S	HMDB0012315	870.5483222	10.28015	↑
34	PE(20:0/18:3(9Z,12Z,15Z))	C43H80NO8P	HMDB0009227	790.5408607	10.87353333	↓
35	12_KETE	C20H30O3	HMDB0013633	317.211439	10.99723333	↑
36	16‐Hydroxyhexadecanoic acid	C16H32O3	HMDB0006294	317.2115701	11.61945	↓
37	Docosahexaenoic acid	C22H32O2	HMDB0002183	327.2326495	12.73883333	↑
38	(2′ E,4′Z,7′Z,8E)_Colnelenic acid	C18H28O3	HMDB0030996	629.4549201	12.93863333	↓
39	DG(15:0/22:5(7Z,10Z,13Z,16Z,19Z)/0:0)	C40H68O5	HMDB0007091	627.4984601	13.79445	↓
40	LysoPC(17:0/0:0)	C25H52NO7P	HMDB0012108	554.3451641	11.52316667	↓
41	LysoPC(18:1(9Z)/0:0)	C26H52NO7P	HMDB0002815	566.3451239	11.05395	↑
42	LysoPE(18:2(9Z,12Z)/0:0)	C23H44NO7P	HMDB0011507	476.277269	10.4382	↓
43	Kinetensin 4‐7	C26H37N9O6	HMDB0012986	592.2640484	10.45195	↑
44	PE(22:5(7Z,10Z,13Z,16Z,19Z)/16:0)	C43H76NO8P	HMDB0009649	764.5268842	10.85291667	↑
45	Allylestrenol	C21H32O	HMDB0015500	345.242761	11.8056	↓
46	Avocadene	C17H34O3	HMDB0031042	267.2318903	13.40783333	↑
47	Adrenic acid	C22H36O2	HMDB0002226	331.2633988	13.6364	↑
48	PC(18:0/P_16:0)	C42H84NO7P	HMDB0008060	780.5662762	15.48383333	↑
49	Heptadecanoic acid	C17H34O2	HMDB0002259	269.2474664	14.4011	↑
50	2‐Dodecylbenzenesulfonic acid	C18H30O3S	HMDB0031031	325.1835593	13.79445	↑
51	6,7‐Dihydro‐4‐(hydroxymethyl)‐2‐(p‐hydroxyphenethyl)‐7‐methyl‐5H‐2‐pyrindinium	C18H22NO2+	HMDB0033483	265.1468937	13.78758333	↑
52	N‐Undecanoylglycine	C13H25NO3	HMDB0013286	242.1752641	13.77385	↓
53	LysoPE(0:0/24:6(6Z,9Z,12Z,15Z,18Z,21Z))	C29H48NO7P	HMDB0011499	552.3085608	10.38323333	↓
54	LysoPC(O_18:0/0:0)	C26H56NO6P	HMDB0011149	554.3827441	9.472233333	↓
55	L‐Glyceric acid	C3H6O4	HMDB0006372	151.0250207	1.236283333	↑
56	Xanthosine	C10H12N4O6	HMDB0000299	283.0673341	2.428966667	↑
57	Inosine L	C10H12N4O5	HMDB0000195	267.0722339	1.719116667	↑
58	Oxidized glutathione	C20H32N6O12S2	HMDB0003337	611.1451283	1.1469	↑
59	Dithianon	C14H4N2O2S2	HMDB0031780	316.9468431	0.6846	↓

### 3.7 Enrichment results of serum metabolic pathways

**FIGURE 7:**
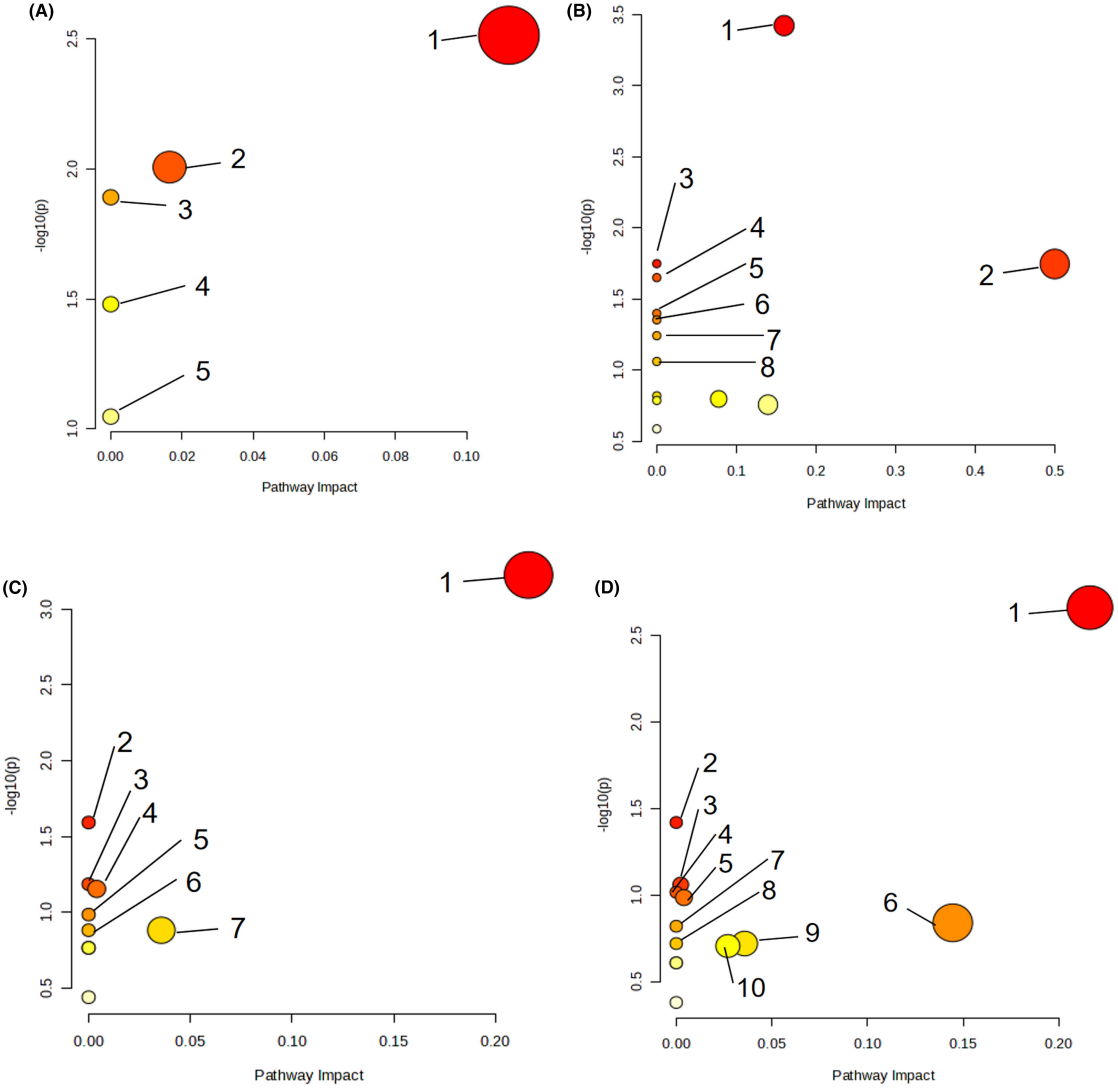
Enrichment Results of Serum Metabolic Pathways. (A) NC group versus NAFLD group (ESI^+^). 1: Glycerophospholipid metabolism; 2: Purine metabolism; 3: Linoleic acid metabolism; 4: α‐linolenic acid metabolism; 5: Arachidonic acid metabolism. (B) NAFLD group versus BL group (ESI^+^). 1: Glycerophospholipid metabolism; 2: Aminoacyl‐tRNA biosynthesis; 3: Biosynthesis of phenylalanine, tyrosine, and tryptophan; 4: Linoleic acid metabolism; 5: Ubiquinone biosynthesis; 6: Phenylalanine metabolism; 7: α‐linolenic acid metabolism; 8: Arachidonic acid metabolism. (C) NC group versus NAFLD group (ESI^−^). 1: Glycerophospholipid metabolism; 2: Linoleic acid metabolism; 3: α‐linolenic acid metabolism; 4: Glycosyl phosphatidylinositol (GPI) anchored biosynthesis; 5: Sphingolipid metabolism; 6: Folate biosynthesis; 7: Galactose metabolism. (D) NAFLD group versus BL group (ESI^−^). 1: Glycerophospholipid metabolism; 2: Linoleic acid metabolism; 3: Purine metabolism; 4: α‐linolenic acid metabolism; 5: Glycosyl phosphatidylinositol (GPI) anchored biosynthesis; 6: Arachidonic acid metabolism; 7: Sphingolipid metabolism; 8: Folate biosynthesis; 9: Galactose metabolism; 10: Glutathione metabolism.

Import the obtained differential metabolites into MetaboAnalyst 5.0 (http://www.metaboanalyst.ca) website for metabolite pathway enrichment analysis. The *X*‐axis represents VIP values, and the *Y*‐axis represents P‐values. BL‐19 regulates multiple metabolic pathways in NAFLD mice, including lipid metabolism, inflammation metabolism, and others such as glycerophospholipid metabolism, arachidonic acid metabolism, sphingolipid metabolism, glutathione metabolism, and peanut oil metabolism (Figure 7).

## 4 DISCUSSION

*Bifidobacterium* is a type of bacteria that does not require oxygen to survive and produces lactic acid (He et al., 2023). It is beneficial for the human gut and helps maintain overall health (Derrien et al., 2022). *B. longum* is the most common species of *Bifidobacterium* found in both infants and adults (Saturio et al., 2021; Sims & Tannock, 2020). It has been recognized as safe by regulatory authorities and is approved for use in food and dietary supplements in China (Skrzydło‐Radomańska et al., 2023; Yao et al., 2021). *B. longum* has various health benefits, including inhibiting the growth of harmful bacteria in the gut, reducing triglyceride and cholesterol levels in the blood, and having antioxidant, antiaging, and immune‐enhancing effects. Our research findings indicate that *B. longum* BL‐19 can effectively prevent weight gain and reduce total energy intake in mice with NAFLD, although it does not significantly affect serum triglyceride and cholesterol levels.

After administering *B. longum* BL‐19 orally to mice, the analysis of their gut microbiota revealed a significant increase in the abundance of butyrate‐producing bacteria like *Oscillospira* and *Coprococcus* in their intestines. Additionally, UPLC‐MS/MS detection confirmed a notable rise in butyrate concentration in the mice's feces. Butyrate is a SCFAs that is produced by specific anaerobic bacteria during the fermentation of dietary fiber in the colon (Ishizuka et al., 2023). It is commonly found in the body as sodium salt (Ashaolu et al., 2021; Nogal et al., 2021; van der Hee & Wells, 2021). Sodium butyrate, a typical histone deacetylase inhibitor (HDACi), has anti‐inflammatory effects by inhibiting acetylase activity (Wang et al., 2023). Numerous studies have demonstrated that sodium butyrate can increase leptin secretion in animals, leading to appetite suppression, increased energy expenditure, and a slower rate of weight gain caused by a high‐fat diet (Jiao et al., 2021; Kushwaha et al., 2022; Peng et al., 2023). We speculate that the inhibition of rapid weight gain and total energy intake in NAFLD mice by *B. longum* BL‐19 may be attributed to this mechanism.

Butyrate has an important biological function in reducing inflammation. Sodium butyrate has been found to inhibit the activation of macrophages and the release of proinflammatory cytokines like interleukins, nitric oxide synthase, and cyclooxygenase‐2 (Han et al., 2020; Siddiqui & Cresci, 2021; Thaweesest et al., 2022). Patients with hyperlipidemia and insulin resistance often exhibit abnormal levels of inflammatory factors, such as IL‐1β and TNF‐α in their adipose tissue and serum compared to healthy individuals (Cheon & Song, 2022; Gasmi et al., 2021).

The abnormal levels of inflammatory factors further lead to oxidative stress, causing damage to tissues and DNA, ultimately contributing to the development of chronic diseases and cancer (Fishbein et al., 2021; Hajam et al., 2022; Jomova et al., 2023). In the study, we discovered that *B. longum* BL‐19 effectively suppresses the expressions of NLRP3 and TNF‐α in NAFLD mice liver, while simultaneously increasing the IL‐10 expression, thereby exerting a significant anti‐inflammatory effect. Additionally, it enhances the production of the liver's antioxidant factor SOD and inhibits the production of lipid peroxide MDA, demonstrating a substantial inhibitory effect on oxidative stress. We hypothesize that *B. longum* BL‐19 enhances the population of butyrate‐producing bacteria in the intestinal tract, leading to increased production of butyrate. This, in turn, improves inflammation and oxidative stress in the livers of NAFLD mice and inhibits excessive weight gain in mice.

Bile acid is a product of cholesterol metabolism in the liver and is an important substance for regulating fat absorption in the intestines (Zhang et al., 2021). Under normal circumstances, cholesterol in the liver is converted into bile acids by the key enzyme CYP7A1, and the bile acids entering the intestines help with lipid absorption (Yu Cai Lim & Kiat Ho, 2023). Bile acid metabolism is crucial for energy metabolism in the body, and an imbalance in bile acid metabolism can lead to lipid metabolism disorders and changes in the immune environment, which is an important pathological mechanism of NAFLD (Guan et al., 2022).

Bile acid, derived from cholesterol metabolism, plays a significant role in regulating fat absorption in the intestines. Normally, cholesterol in the liver is converted into bile acids by the key enzyme CYP7A1, and these bile acids aid in lipid absorption in the intestines (Yu Cai Lim & Kiat Ho, 2023). The metabolism of bile acid is essential for energy metabolism in the body, and an imbalance in this process can result in lipid metabolism disorders and alterations in the immune environment, which are important pathological mechanisms of NAFLD (Guan et al., 2022).

FXR, an important receptor for bile acids, plays a crucial role in their composition and content (Ku et al., 2012). The activity of FXR can either activate or inhibit the biological effects of bile acids (Radun & Trauner, 2021). Specifically, FXR primarily regulates the synthesis of bile acids by controlling the expression of CYP7A1 (Chiang & Ferrell, 2022). Activation of FXR in liver cells induces the expression of small heterodimer partner (SHP), which acts as an auxiliary inhibitor to suppress the transcriptional activity of liver‐related homolog‐1 (LRH‐1) or hepatocyte nuclear factor 4α (HNF4α), leading to the inhibition of CYP7A1 transcription and subsequent reduction in bile acid synthesis (Panzitt & Wagner, 2021). We observed a significant downregulation of FXR expression in the liver of NAFLD mice, accompanied by a significant upregulation of CYP7A1 expression. This resulted in excessive bile acid synthesis and accumulation in the liver, exacerbating inflammation and liver fibrosis in the mice. Surprisingly, treatment with *B. longum* BL‐19 did not significantly alter the expression of FXR and FGF‐15 in mice, nor did it significantly change the serum TBA content. However, it did significantly increase the expression of CYP7A1. These findings contradict the notion that improving NAFLD symptoms involves inhibiting CYP7A1 expression and bile acid synthesis through the FXR signaling pathway.

We hypothesize that *B. longum* BL‐19 has the ability to enhance butyrate production in the intestines. This, in turn, directly stimulates the expression of CYP7A1 in the liver which could accelerate the reaction that consumes cholesterol and synthesizes bile acids (Guo et al., 2023; Hamada et al., 2020). Some intestinal bacteria possess bile salt hydrolase (BSH), which aids in the conversion of bile acids (Bourgin et al., 2021). In their study, He et al. found that *Bifidobacterium* had a significant impact on the levels of butyrate in the intestines. Additionally, it increased the abundance of bacteria with BSH activity, leading to a greater conversion of conjugated bile acids to free bile acids within the intestinal lumen (He et al., 2021). Due to their lower solubility and absorption efficiency in the intestinal lumen, free bile acids are found in higher quantities in feces compared to conjugated bile acids (Laustiola et al., 1988). Consequently, this leads to an accelerated rate of cholesterol synthesis into bile acids in the liver and an upregulation in the expression of CYP7A1 (Chiang & Ferrell, 2020).

Additional metabolomics research has revealed that *B. longum* BL‐19 has a significant impact on the serum metabolomics of mice with NAFLD. This impact primarily involves multiple metabolic pathways, including lipid metabolism and inflammation metabolism. Uric acid, an endogenous danger signal and activator of the inflammasome, is an independent risk factor for the development of liver cirrhosis (Fernández Rodríguez et al., 2019). Studies have discovered a positive correlation between blood uric acid levels and the severity of NAFLD in patients.

Palmitoyl phosphatidylcholine reflects the level of endogenous lipid synthesis and metabolism. An increase in the level of palmitoyl phosphatidylcholine in the serum indicates enhanced lipid synthesis (Mato et al., 2019). Insulin resistance leads to increased *de novo* synthesis of fat in the liver and inhibits the breakdown of fat in adipose tissue, ultimately resulting in an increase in total fatty acid content in liver cells (Tong et al., 2022). Prolonged excess lipid supply saturates the liver's capacity for fatty acid oxidation and secretion of triglycerides, leading to the accumulation of toxic lipid metabolites through nonoxidative pathways in liver cells (Wang et al., 2019).

NAFLD patients have significantly elevated levels of Lysophosphatidylcholine (LysoPE) in their bodies (Yamamoto et al., 2021). High concentrations of LysoPE in the liver can disrupt mitochondrial integrity and lead to the release of proinflammatory substance cytochrome C from mitochondria, thereby activating oxidative stress and inflammatory responses (Li et al., 2022). NAFLD mice treated with *B. longum* BL‐19 showed a significant decrease in serum LysoPE levels.

Indoleacrylic acid is a tryptophan metabolite produced by bacteria in the intestines. Indoleacrylic acid has significant antioxidant abilities and can eliminate free radicals and prevent oxidative damage (Hendrikx & Schnabl, 2019). Indoleacrylic acid can inhibit macrophage activation and proinflammatory cytokine secretion induced by bacterial lipopolysaccharides (Hendrikx & Schnabl, 2019). In this study, the level of indoleacrylic acid in mouse serum was significantly increased, which is related to the anti‐inflammatory and antioxidant abilities of *B. longum* BL‐19.

Glycerophosphocholine is also a compound with anti‐inflammatory and antioxidant functions. Its concentration in the serum of NAFLD patients is significantly decreased compared to healthy individuals, making it a biomarker for liver damage and fibrosis in NAFLD patients (Beyoğlu & Idle, 2020). In this study, NAFLD mice treated with *B. longum* BL‐19 showed a significant increase in serum glycerophosphocholine levels, indicating that liver damage in mice was inhibited. Stearoylethanolamide is also closely associated with obesity and insulin resistance (Li & Chiang, 2014). When its concentration increases, it can significantly alleviate chronic inflammation and insulin resistance in the body (Mock et al., 2023). In this study, the serum Stearoylethanolamide levels in NAFLD mice were significantly increased, which is also related to the alleviation of chronic inflammation in mice by *B. longum* BL‐19. L‐carnitine has significant antioxidant stress ability, can maintain normal mitochondrial function, and alleviate insulin resistance (Li & Zhao, 2021). Upregulating the serum L‐carnitine levels in NAFLD mice treated with *B. longum* BL‐19 can help improve inflammation and oxidative stress in mice, as well as liver inflammation damage and lipid deposition.

16‐hydroxyhexadecanoic acid is a saturated fatty acid. The concentration of 16‐hydroxyhexadecanoic acid in the blood significantly increases during a high‐fat diet and may further induce disturbances in glucose and lipid metabolism in the human body (Chen et al., 2023). Docosahexaenoic acid is a beneficial medium‐chain fatty acid. Studies have shown that docosahexaenoic acid can inhibit *de novo* lipogenesis in the liver, redirecting fatty acids used for triglyceride synthesis toward β‐oxidation. Docosahexaenoic acid can also upregulate the activity of fatty acid β‐oxidation enzymes in liver mitochondria, ultimately reducing hepatic lipid deposition and circulating triglyceride levels (Calder, 2022). Clinical research has also found that supplementing docosahexaenoic acid to NAFLD patients significantly reduces hepatic lipid accumulation (Valenzuela & Videla, 2020). N‐undecanoylglycine is a glycine derivative primarily involved in fatty acid oxidation and NAFLD patients have significantly elevated levels of N‐undecanoylglycine (Goon et al., 2021). In this study, after treatment with *B. longum* BL‐19, the level of N‐undecanoylglycine in mouse serum was significantly reduced, indicating improved fatty acid oxidation and decreased oxidative stress levels in the body. Inosine has anti‐inflammatory and hepatoprotective functions. It can reduce the levels of alanine aminotransferase (ALT) and aspartate aminotransferase (AST) in the serum of mice with acute liver injury and increase the activity of antioxidant enzymes such as superoxide dismutase (SOD) in the serum (Guo et al., 2021; Morrell et al., 2020). In this study, after treatment with *B. longum* BL‐19, the level of inosine in mouse serum significantly increased, and the inflammatory response and liver damage in mice were alleviated.

## 5 CONCLUSIONS

To summarize, *B. longum* BL‐19 shows promise as a drug for adjunctive treatment of NAFLD. It effectively improves oxidative stress and inflammation in the liver of NAFLD mice, while also reducing lipid deposition. Additionally, *B. longum* BL‐19 increases levels of antioxidant metabolites in the serum of NAFLD mice, as indicated by nontargeted metabolomics results. Furthermore, *B. longum* BL‐19 enhances the production of butyric acid in the mouse gut, as observed through mouse gut microbiota and UPLC‐MS/MS detection. This increase in butyric acid regulates liver CYP7A1 activity and bile acid production, effectively treating inflammation and lipid deposition in the liver of NAFLD mice.

## AUTHOR CONTRIBUTIONS

**Xiajun Zhang:** Conceptualization (equal); data curation (equal); funding acquisition (equal); writing – original draft (equal). **Jingwen Xu:** Data curation (equal); formal analysis (equal); methodology (equal). **Xueyun Dong:** Conceptualization (equal); data curation (equal); visualization (equal). **Jiajun Tang:** Methodology (equal); visualization (equal); writing – original draft (equal). **Yan Xie:** Data curation (equal); formal analysis (equal). **Jie Yang:** Data curation (equal); formal analysis (equal); investigation (equal). **Limin Zou:** Methodology (equal); resources (equal); software (equal). **Liang Wu:** Conceptualization (equal); data curation (equal); investigation (equal); project administration (equal); supervision (equal); visualization (equal); writing – original draft (equal); writing – review and editing (equal). **Jilong Fan:** Conceptualization (equal); data curation (equal); formal analysis (equal); funding acquisition (equal); project administration (equal); supervision (equal); visualization (equal); writing – original draft (equal); writing – review and editing (equal).

## CONFLICT OF INTEREST STATEMENT

The authors declare that they have no conflict of interest.

## ETHICS STATEMENT

The study was conducted in accordance with the National Institutes of Health guide for the care and use of Laboratory animals (NIH Publications No. 8023, revised 1978) and approved by the Ethics Committee of the Jiangsu University (protocol code UJS‐IACUC‐AP‐2021032009 and date of approval: January 2021).

## Data Availability

The raw data supporting the conclusions of this article will be made available by the authors.
